# Efficacy and Safety of Biosimilar SAR342434 Insulin Lispro in Adults with Type 2 Diabetes, Also Using Insulin Glargine: SORELLA 2 Study

**DOI:** 10.1089/dia.2017.0281

**Published:** 2018-01-01

**Authors:** Karl-Michael Derwahl, Timothy S. Bailey, Karin Wernicke-Panten, Lin Ping, Suzanne Pierre

**Affiliations:** ^1^ Institut für Klinische Forschung und Entwicklung (IKFE) Berlin GmbH, Berlin, Germany.; ^2^ AMCR Institute, Escondido, California.; ^3^ Sanofi-Aventis Deutschland GmbH, Frankfurt, Germany.; ^4^ Sanofi, Bridgewater, New Jersey.; ^5^ Sanofi, Paris, France.

**Keywords:** Biosimilar, Insulin antibodies, Immunogenicity, Insulin lispro, SORELLA 2.

## Abstract

***Background:*** SAR342434 (SAR-Lis) is a biosimilar (follow-on) of insulin lispro (U100; Humalog^®^; Ly-Lis). This study aimed to show similar efficacy, safety, and immunogenicity of SAR-Lis versus Ly-Lis in adult patients with type 2 diabetes mellitus (T2DM) treated with multiple daily injections, while using insulin glargine (GLA-100; Lantus^®^) as basal insulin.

***Methods:*** SORELLA 2 was a 6-month, randomized, open-label, Phase 3 study (NCT02294474). Insulin doses were adjusted to achieve fasting and 2-h postprandial glucose targets according to American Diabetes Association guidelines. Primary endpoint was the HbA_1c_ change from baseline to week 26 (tested for noninferiority of SAR-Lis vs. Ly-Lis with a margin of 0.3%). Secondary endpoints included fasting plasma glucose (FPG), seven-point self-monitored plasma glucose (SMPG) profiles, hypoglycemic events, treatment-emergent adverse events (TEAEs), and anti-insulin antibodies (AIA).

***Results:*** A total of 505 patients were randomized (1:1) to multiple daily injections of SAR-Lis (*n* = 253) or Ly-Lis (*n* = 252) plus once-daily GLA-100. Least square (LS) mean (standard error) change in HbA_1c_ from baseline to week 26 was similar in both treatment groups (SAR-Lis, −0.92% [0.051] and Ly-Lis, −0.85% [0.051]). Noninferiority at prespecified 0.3% noninferiority margin was demonstrated (LS mean difference of SAR-Lis vs. Ly-Lis: −0.07% [95% CI: −0.215 to 0.067]) as was inverse noninferiority. Similar changes in FPG, seven-point SMPG profiles, including postprandial glucose excursions and mean glucose over 24 h, and insulin dosages were observed in the two groups. Hypoglycemia, TEAEs, and AIA (incidence and prevalence) did not differ between groups.

***Conclusions:*** Results from this controlled study in patients with T2DM also using GLA-100 support similar efficacy and safety (including immunogenicity) of SAR-Lis and Ly-Lis.

## Introduction

Patients with type 2 diabetes mellitus (T2DM) on oral antihyperglycemic treatment, who require insulin to achieve good glycemic control, most often start insulin therapy with a basal insulin, such as insulin glargine (GLA-100; Lantus^®^), to control fasting blood glucose levels. If adequate glycemic control cannot be obtained by controlling fasting blood glucose alone, it becomes necessary to also control prandial glucose levels. Among the options is to add a short- or rapid-acting insulin. According to the American Diabetes Association (ADA), recommended therapy for T2DM consists of matching the prandial insulin dose to premeal blood glucose, carbohydrate intake, and anticipated activity.^1^ For many patients (especially if hypoglycemia is a problem), use of insulin analogs is recommended.^2^ The use of rapid-acting insulin analogs, such as insulin lispro, insulin aspart, or insulin glulisine, also facilitates flexible dosing in these insulin treatment regimens.

Insulin lispro differs from human insulin in that the amino acid proline at position B28 is replaced by lysine and the lysine in position B29 is replaced by proline. This modification does not alter insulin receptor binding, but blocks the formation of dimers and hexamers. As a consequence, higher amounts of subcutaneous monomers are available for rapid absorption. This enables a shorter waiting period after injection before starting the meal. Insulin lispro is the active ingredient of Humalog^®^ (Ly-Lis; Lilly).^3^ Humalog was the first rapid-acting insulin approved and marketed in the EU, the US, and many other countries worldwide, and is used in the treatment of both patients with type 1 diabetes mellitus (T1DM) and T2DM.

SAR342434 (SAR-Lis; insulin lispro; Sanofi) has been developed as a biosimilar (follow-on) biological medicinal product to Humalog U100 in accordance with the relevant US and EU guidelines, including the EU guidelines for similar medicinal products containing recombinant human insulin and insulin analogs, as well as product-specific guidelines.^4–8^ SAR-Lis was shown to be highly similar to Ly-Lis through physicochemical analyses and in vitro and in vivo nonclinical studies. Similar pharmacokinetic (PK) exposure and pharmacodynamic (PD) activity were demonstrated for SAR-Lis to both Ly-Lis approved in the EU and Ly-Lis approved in the US, as well as between Ly-Lis US and Ly-Lis EU in a PK/PD study in patients with T1DM using the euglycemic clamp technique.^9,10^ This report presents the results of a multinational, open-label, randomized, controlled Phase 3 study (SORELLA 2) comparing the efficacy and safety of SAR-Lis and the reference product Ly-Lis (100 U/mL) in patients with T2DM, also using insulin glargine (GLA-100).

## Methods

The study was approved by relevant review boards/ethics committees, and was performed in accordance with the Declaration of Helsinki and the International Conference on Harmonisation guidelines. All participants provided written informed consent before participation.

### Study patients

Eligible patients were of the legal age of adulthood with HbA_1c_ ≥6.5% and ≤10%, T2DM diagnosed for at least 12 months, and treated with Humalog/Liprolog^®^ (insulin lispro) or NovoLog^®^/NovoRapid^®^ (rapid-acting insulin aspart) at least thrice daily before each meal and GLA-100 as basal insulin in the 6 months before the screening visit. Noninsulin antihyperglycemic background therapy taken at a stable dose for at least 3 months before the screening visit was permitted. Excluded were patients with body mass index (BMI) ≥40 kg/m^2^, the use of noninsulin injectable peptides (e.g., GLP1-receptor agonists or other peptides), use of continuous subcutaneous insulin infusion, history of severe hypoglycemia requiring treatment by emergency room admission, or poor metabolic control requiring hospitalization, all within the last 6 months before screening. Also excluded were women of childbearing potential not protected by a highly effective contraceptive method and patients with unstable proliferative diabetic retinopathy or any other rapidly progressive diabetic retinopathy, or macular edema likely to require treatment (e.g., laser, surgical treatment, or injectable drugs) during the study.

### Study design

SORELLA 2 (NCT02294474) was a multicenter, 6-month, randomized, open-label, two-arm parallel-group, Phase 3 study comparing SAR-Lis with Ly-Lis in adults with T2DM also using GLA-100 as basal insulin. The study consisted of a screening period (up to 2 weeks), a 26-week treatment period, and a 1-day safety follow-up (Supplementary Fig. S1; Supplementary Data are available online at http://online.liebertpub.com/doi/suppl/10.1089/dia.2017.0281). Clinical visits were scheduled for screening, randomization (day 1), weeks 4, 8, 12, 20, and 26 (endpoint). After the screening period, 480 patients were planned to be randomized 1:1 to receive either SAR-Lis or Ly-Lis in addition to the once-daily Gla-100. The randomization was stratified by HbA_1c_ obtained at the screening visit (<8.0%, ≥8.0%) and prior use of insulin lispro (Yes, No). The randomization and the treatment kit allocation were performed centrally by an interactive voice response system/interactive web response system. The comparator drug in the study was Ly-Lis. Patients randomized to Ly-Lis received US- or EU-approved Ly-Lis, depending on the location of their study site. Based on the similarity between Ly-Lis US and Ly-Lis EU shown in physicochemical analyses, nonclinical studies, and the PK/PD study,^9^ data from both insulins were pooled in the comparator group of this study.

Study medications were dispensed on day 1, and weeks 4, 8, 12, and 20. Self-monitored plasma glucose (SMPG) and insulin dose data were obtained from the patient's diary at each visit when compliance was checked by reviewing the patient's diary and counting/collecting used and unused pens. Starting dose of SAR-Lis or Ly-Lis was a unit-to-unit conversion from the Humalog/Liprolog or Novolog/NovoRapid dose used before the trial. SAR-Lis or Ly-Lis was administered subcutaneously (SC), immediately before meal intake using insulin pens. Occasional postprandial injections soon after meal intake were permitted if deemed necessary and if allowed by the national product label for Ly-Lis. Mealtime insulin dose could be adjusted to achieve a target range for 2-h postprandial plasma glucose (PG) of 120–160 mg/dL (6.7–8.9 mmol/L). The starting dose of GLA-100 was the same as the prestudy dose. GLA-100 was injected SC once daily at the same time throughout the study, and dose adjustments were made to achieve a fasting, prebreakfast PG of 80–130 mg/dL (4.4–7.2 mmol/L). No formal titration algorithm was recommended for basal insulin; patients were instructed to use dosage self-adjustment of rapid-acting insulin analogs according to local guidelines to achieve target glucose, while avoiding hypoglycemia.

### Efficacy, safety, and immunogenicity assessments

HbA_1c_ and fasting plasma glucose (FPG) were determined in a central laboratory blinded for treatment (Covance, Indianapolis, IN) at screening (HbA_1c_ only), baseline, week 12, and week 26. Seven-point SMPG profiles (preprandial and 2-h postprandial after breakfast, lunch, and dinner, and at bedtime) were to be performed on at least 2 days in the week before baseline, week 12, and week 26, measured in a single, 24-h period using the Bluetooth-enabled glucometer “myGlucoHealth” (Entra Health Systems, San Diego, CA). SMPG results, hypoglycemic events, and insulin doses were recorded by the patients in a paper diary and manually entered in the e-CRF by the investigator. Adverse events (AEs), including hypersensitivity events and injection site reactions, were documented at each visit. Further safety monitoring included hematology and clinical chemistry, as well as body weight.

Blood samples for anti-insulin antibodies (AIA) determination were to be drawn at least 8 h after the last administration of mealtime insulin on day 1, at weeks 4 and 12, and at the end of treatment at week 26. AIA were determined employing a validated radio immunoprecipitation assay in a central laboratory blinded for treatment. The assay was validated in agreement with recent literature.^10^ An Allergic Reaction Assessment Committee (ARAC) consisted of four experts, three of whom were board certified in allergy and clinical immunology and reviewed all hypersensitivity reactions reported on a specific allergic reaction AE form or identified by Medical Dictionary for Regulatory Activities (MedDRA) search, and one of whom was certified in diabetes mellitus and reviewed all cases of potential effects of AIA on efficacy (insulin dose, HbA_1c_) and safety (hypoglycemia, injection site, and hypersensitivity reaction).

### Study objectives

The primary objective of the study was to demonstrate noninferiority of SAR-Lis versus Ly-Lis in terms of changes in HbA_1c_ from baseline to week 26 at a noninferiority margin of 0.3% in patients with T2DM, also using GLA-100. Secondary objectives were to assess the immunogenicity of SAR-Lis and Ly-Lis in terms of positive/negative status and antibody titers at baseline and during the course of the study; to assess the relationship of AIA with efficacy and safety; to assess the efficacy of SAR-Lis and Ly-Lis in terms of patients reaching target HbA_1c_ <7.0% and ≤6.5%, FPG, SMPG profiles, and insulin dose; and to assess the safety of SAR-Lis and Ly-Lis.

### Study endpoints

Efficacy endpoints included change from baseline to week 26 in HbA_1c_ (primary endpoint), FPG, 24-h PG concentration from seven-point SMPG profiles and postprandial PG excursions (difference between 2-h postprandial and preprandial PG values from seven-point SMPG profiles), as well as the proportion of patients reaching target HbA_1c_ <7.0% and ≤6.5% at week 26. Safety endpoints included the percentage of patients reporting at least one hypoglycemic event, hypoglycemic event rates, the occurrence of treatment-emergent AEs (TEAEs), including hypersensitivity and injection site reactions, and change in body weight and clinical laboratory and hematology parameters. Hypersensitivity events and injection site reactions were identified using specific MedDRA codes. TEAEs were defined as events that occurred, worsened, or became serious from first investigational medicinal product (IMP) intake up to 1 day after last IMP intake.

Hypoglycemia was categorized based on the ADA definitions.^11^ Documented symptomatic hypoglycemia was an event during which typical symptoms of hypoglycemia were accompanied by a measured PG concentration ≤3.9 mmol/L (≤70 mg/dL); those with PG <3.0 mmol/L (<54 mg/dL) were also analyzed. Nocturnal hypoglycemia was defined as any hypoglycemia that occurred between 00:00 and 05:59 a.m. hours. Severe hypoglycemia was an event that required assistance of another person to actively administer carbohydrate, glucagon, or other resuscitative actions. Severe hypoglycemia associated with seizure, unconsciousness, or coma was also to be reported as a serious AE.

Immunogenicity was assessed by incidence (patients with newly positive postbaseline [treatment induced] or with ≥4-fold increase in titer [treatment boosted], i.e., patients with treatment-emergent AIA) and prevalence (patients with at least one positive sample at baseline or postbaseline) of AIA, and using sample status, titer, and cross-reactivity to human insulin, insulin glargine, and insulin glargine M1 metabolite.

### Statistical analysis

Efficacy analyses were performed in the intent-to-treat (ITT) population, which included all randomized patients, irrespective of compliance with the study protocol and procedures. Noninferiority on the primary efficacy endpoint (change in HbA_1c_ from baseline to week 26) was tested at the prespecified 0.3% margin, with α level of 0.025 (one sided). If noninferiority of SAR-Lis over Ly-Lis was demonstrated, using a hierarchical step-down testing procedure, the inverse noninferiority (of Ly-Lis over SAR-Lis) was tested. Least square (LS) means were obtained from a mixed-effect model for repeated measures using all available postbaseline HbA_1c_ data, adjusted on treatment, randomization strata, visit, treatment-by-visit interaction, baseline, and baseline-by-visit interaction, and with an unstructured correlation matrix to model the within-patient errors. Parameters were estimated using restricted maximum likelihood method with the Newton-Raphson algorithm, and denominator degrees of freedom were estimated using Satterthwaite's approximation.

A sample size of 480 randomized patients (240 patients/arm) was considered sufficient to ensure that the upper bound of the two-sided 95% confidence interval (CI) for the adjusted mean difference between SAR-Lis and Ly-Lis on HbA_1c_ change from baseline to week 26 would not exceed 0.3% HbA_1c_ with at least 90% power. This calculation assumed a common standard deviation of 1.0% and a true difference in HbA_1c_ between the treatment groups of zero. All other efficacy, safety, and immunogenicity analyses were descriptive.

Safety analyses were based on the safety population, defined as all patients randomized and exposed to at least one dose of SAR-Lis or Ly-Lis, regardless of the amount of treatment administered. The AIA analyses were based on the AIA population, defined as all patients from the safety population with at least one AIA sample available for analysis during the 6-month on-treatment period (from first IMP intake up to 1 day after last IMP intake). All analyses were conducted using SAS Enterprise Guide version 5.1.

## Results

### Patient disposition and baseline characteristics

**FIG. 1.**
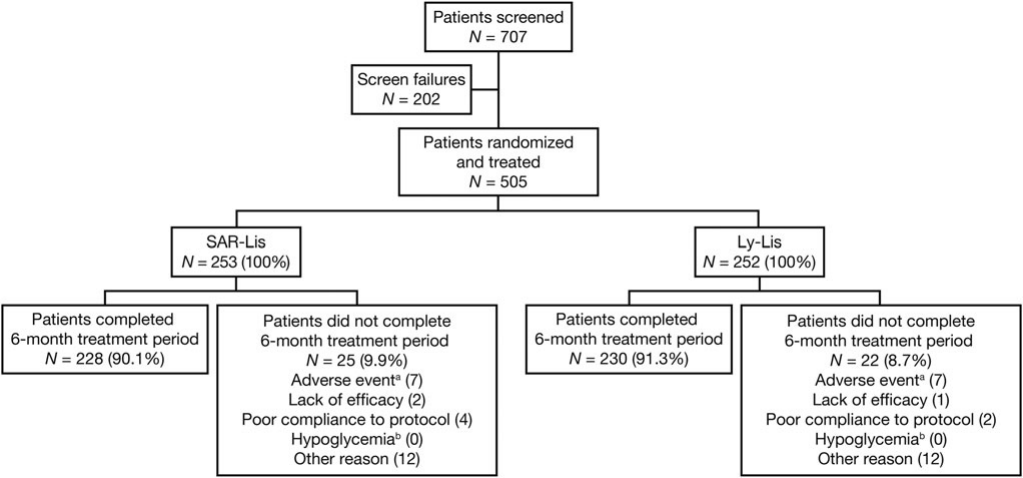
Patient disposition.

A total of 707 patients were screened for the study; 202 (28.6%) were screen failures (Fig. 1). The most common reason for screen failure was HbA_1c_ <6.5% or >10% (76 patients [10.7%]). Five hundred five patients were randomized and treated (ITT and safety population): 253 patients in the SAR-Lis group and 252 patients in the Ly-Lis group. Two hundred twenty-eight patients (90.1%) in the SAR-Lis group and 230 (91.3%) in the Ly-Lis group completed the treatment period. A similar number of patients in each group discontinued the study treatment prematurely (SAR-Lis, 25 [9.9%], and Ly-Lis, 22 [8.7%]). The most common reasons for treatment discontinuation were “Other reasons,” which included patient decision or consent withdrawal, and “Adverse events.”

Demographic and baseline characteristics were well balanced between treatment groups (Table 1). The mean age of the randomized population was 62.5 years, and more than 40% of the population was 65 years or older. Most patients (93%) were overweight or obese (BMI ≥25 to <30 kg/m^2^ or ≥30 kg/m^2^), and the mean BMI was 32.2 kg/m^2^. The mean duration of diabetes was 17.1 years, with 80.8% of patients with a duration ≥10 years. Overall, 19.8% of the population had a moderate impairment (estimated glomerular filtration rate [eGFR] ≥30 and <60 mL/min/1.73 m^2^) and four patients (0.8% of all patients) had a severe impairment of renal function (eGFR <30 mL/min/1.73 m^2^). Previous mealtime insulin use was Humalog/Liprolog (51.4%), NovoLog/NovoRapid (48.2%), or both (0.4%). Mean HbA_1c_ value was 8.0% (64 mmol/mol).

**Table 1.** Demographic and Baseline Characteristics—Randomized Population

	*SAR-Lis (*N* = 253)*	*Ly-Lis (*N* = 252)*	*All (*N* = 505)*
Age (years), mean (SD) [*n*]	62.1 (9.4) [253]	62.8 (8.9) [252]	62.5 (9.1) [505]
Age groups, *n* (%)			
<65	144 (56.9)	137 (54.4)	281 (55.6)
≥65 to <75	89 (35.2)	93 (36.9)	182 (36.0)
≥75	20 (7.9)	22 (8.7)	42 (8.3)
Male gender, *n* (%)	136 (53.8)	132 (52.4)	268 (53.1)
Race, *n* (%)			
Caucasian/White	228 (90.1)	218 (86.5)	446 (88.3)
Black	14 (5.5)	17 (6.7)	31 (6.1)
Asian/Oriental	11 (4.3)	16 (6.3)	27 (5.3)
Other	0	1 (0.4)	1 (0.2)
Ethnicity, *n* (%)			
Hispanic	43 (17.0)	47 (18.7)	90 (17.8)
Not Hispanic	210 (83.0)	205 (81.3)	415 (82.2)
Regions, *n* (%)			
United States	122 (48.2)	120 (47.6)	242 (47.9)
Western Europe	32 (12.6)	37 (14.7)	69 (13.7)
Eastern Europe	67 (26.5)	58 (23.0)	125 (24.8)
Rest of the world	32 (12.6)	37 (14.7)	69 (13.7)
Region-approved Humalog^®^, *n* (%)			
US-approved Humalog	122 (48.2)	120 (47.6)	242 (47.9)
EU-approved Humalog	131 (51.8)	132 (52.4)	263 (52.1)
Baseline weight (kg), mean (SD) [*n*]	92.2 (17.5) [253]	91.2 (17.4) [252]	91.7 (17.4) [505]
Baseline BMI (kg/m^2^), mean (SD) [*n*]	32.3 (4.8) [253]	32.1 (4.8) [252]	32.2 (4.8) [505]
Baseline BMI categories, *n* (%)			
<25	17 (6.7)	18 (7.1)	35 (6.9)
≥25 to <30	62 (24.5)	72 (28.6)	134 (26.5)
≥30	174 (68.8)	162 (64.3)	336 (66.5)
Baseline estimated GFR (mL/min/1.73 m^2^), mean (SD) [*n*]	77.29 (22.89) [253]	78.48 (23.66) [252]	77.89 (23.26) [505]
Baseline estimated GFR categories (mL/min/1.73 m^2^), *n* (%)			
≥90	69 (27.3)	67 (26.6)	136 (26.9)
≥60 to <90	130 (51.4)	135 (53.6)	265 (52.5)
≥30 to <60	51 (20.2)	49 (19.4)	100 (19.8)
<30	3 (1.2)	1 (0.4)	4 (0.8)
Randomization strata of screening HbA_1c_ categories, *n* (%)			
<8%	105 (41.5)	104 (41.3)	209 (41.4)
≥8%	148 (58.5)	148 (58.7)	296 (58.6)
Randomization strata of prior use of Humalog, *n* (%)			
Yes	155 (61.3)	155 (61.5)	310 (61.4)
No	98 (38.7)	97 (38.5)	195 (38.6)
Duration of T2DM (years), mean (SD) [*n*]	16.60 (7.93) [253]	17.52 (8.67) [252]	17.06 (8.31) [505]
Duration of T2DM categories (years), *n* (%)			
<10	50 (19.8)	47 (18.7)	97 (19.2)
≥10	203 (80.2)	205 (81.3)	408 (80.8)
Age at onset of T2DM (years), mean (SD) [*n*]	46.0 (10.1) [253]	45.8 (10.2) [252]	45.9 (10.1) [505]
Duration of basal bolus insulin treatment (years), mean (SD) [*n*]	7.10 (5.67) [247]	7.99 (6.76) [243]	7.54 (6.24) [490]
Duration of mealtime insulin treatment in patient life (years), mean (SD) [*n*]	6.43 (5.54) [250]	7.17 (6.33) [247]	6.80 (5.95) [497]
Previous basal insulin type, *n* (%)			
Insulin glargine	253 (100)	251 (99.6)	504 (99.8)
Duration of insulin glargine treatment (years), mean (SD) [*n*]	5.75 (4.62) [253]	5.97 (4.69) [252]	5.86 (4.65) [505]
Previous mealtime insulin type, *n* (%)			
Humalog/Liprolog^®^	133 (52.6)	126 (50.2)	259 (51.4)
NovoLog^®^/NovoRapid^®^	119 (47.0)	124 (49.4)	243 (48.2)
Both Humalog/Liprolog and NovoLog/NovoRapid	1 (0.4)	1 (0.4)	2 (0.4)
Duration of previous treatment with Humalog/Liprolog (years), mean (SD) [*n*]	5.36 (5.29) [134]	4.64 (4.55) [127]	5.01 (4.95) [261]
Duration of previous treatment with NovoLog/NovoRapid (years), mean (SD) [*n*]	4.51 (4.41) [120]	5.72 (5.40) [125]	5.13 (4.97) [245]
Baseline HbA_1c_,%, mean (SD) [*n*]	7.99 (0.87) [253]	8.03 (0.91) [252]	8.01 (0.89) [505]

BMI, body mass index; GFR, glomerular filtration rate; Ly-Lis, Humalog insulin lispro; SAR-Lis, SAR342434 insulin lispro; SD, standard deviation; T2DM, type 2 diabetes mellitus.

### Efficacy

**FIG. 2.**
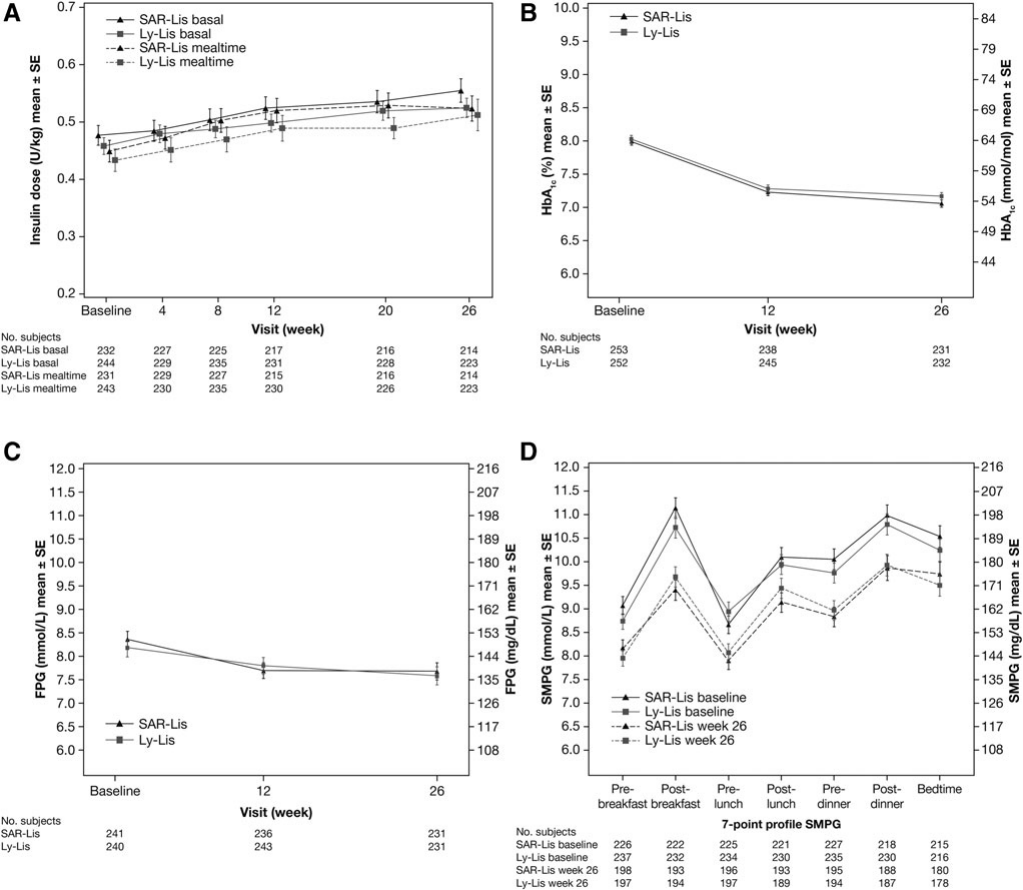
Mean (SE) insulin dose **(A)**, HbA_1c_ **(B)**, fasting plasma glucose **(C)**, and seven-point SMPG time profiles **(D)**. Data for (**A)** is from the safety population, for **(B–D)**, the ITT population. For **(A)**, baseline is defined as the mean of daily doses available in the week before the first injection of SAR-Lis or Ly-Lis. ITT, intent-to-treat; Ly-Lis, Humalog^®^ insulin lispro; SAR-Lis, SAR342434 insulin lispro; SE, standard error; SMPG, self-monitored plasma glucose.

Changes in the daily mealtime insulin doses were small and similar in both groups over the 26-week treatment period and occurred mainly within the first 4 to 8 weeks of treatment (Fig. 2A). Mean (SD) dose at baseline was 0.449 (0.294) U/kg/day for SAR-Lis and 0.433 (0.315) U/kg/day for Ly-Lis, and at 26 weeks was 0.524 (0.329) U/kg/day and 0.512 (0.420) U/kg/day, respectively (Table 2). The change from baseline to week 26 was 0.087 (0.209) U/kg/day for SAR-Lis and 0.080 (0.248) U/kg/day for Ly-Lis. Baseline doses of GLA-100 were also similar in both groups. A modest increase of the basal insulin daily doses was also observed in both treatment groups over the 26-week treatment period. Mean (SD) dose at baseline was 0.477 (0.265) U/kg/day for the SAR-Lis group and 0.458 (0.239) U/kg/day for the Ly-Lis group, and change from baseline was 0.082 (0.133) U/kg/day for SAR-Lis and 0.071 (0.122) U/kg/day for Ly-Lis (Table 2).

**Table 2.** Summary of Insulin Doses and Glycemic Control

	*SAR-Lis*	*Ly-Lis*
Insulin dose—safety population		
Mealtime insulin, U/kg, mean (SD)		
Baseline [*n*]	0.449 (0.294) [231]	0.433 (0.315) [243]
Week 26 [*n*]	0.524 (0.329) [214]	0.512 (0.420) [223]
Change from baseline [*n*]	0.087 (0.209) [197]	0.080 (0.248) [218]
Basal insulin, U/kg, mean (SD)		
Baseline [*n*]	0.477 (0.265) [232]	0.458 (0.239)[244]
Week 26 [*n*]	0.555 (0.303) [214]	0.525 (0.262) [223]
Change from baseline [*n*]	0.082 (0.133) [196]	0.071 (0.122) [218]
Glycemic control—ITT population (patients included in the MMRM analysis)		
HbA_1c_, %-units; mean (SD)		
Baseline [*n*]	8.00 (0.86) [239]	8.03 (0.91) [246]
Week 26 [*n*]	7.06 (0.85) [231]	7.16 (0.88) [232]
LS mean change (SE) from baseline [*n*]	−0.92 (0.051) [239]	−0.85 (0.051) [246]
LS mean difference (SE) [95% CI]	−0.07 (0.072) [−0.215 to 0.067]	
FPG, mmol/L; mean (SD)		
Baseline [*n*]	8.35 (2.67) [228]	8.18 (2.80) [235]
Week 26 [*n*]	7.65 (2.71) [220]	7.53 (2.81) [220]
LS mean change (SE) from baseline [*n*]	–0.62 (0.176) [228]	−0.67 (0.176) [228]
LS mean difference (SE) [95% CI] vs. Ly-Lis	0.06 (0.249) [−0.430 to 0.547]	
Postprandial glucose excursion from SMPG, mmol/L; mean (SD)		
Breakfast		
Baseline [*n*]	1.96 (3.27) [194]	1.82 (3.46) [204]
Week 26 [*n*]	1.30 (3.17) [171]	1.77 (3.14) [184]
LS mean change (SE) from baseline [*n*]	−0.72 (0.236) [194]	−0.23 (0.228) [204]
LS mean difference (SE) [95% CI] vs. Ly-Lis	−0.48 (0.328) [−1.127 to 0.164]	
Lunch		
Baseline [*n*]	1.71 (3.36) [195]	1.11 (3.68) [200]
Week 26 [*n*]	1.42 (3.52) [170]	1.33 (3.26) [174]
LS mean change (SE) from baseline [*n*]	0.06 (0.255) [195]	0.11 (0.250) [200]
LS mean difference (SE) [95% CI] vs. Ly-Lis	−0.05 (0.357) [−0.749 to 0.655]	
Dinner		
Baseline [*n*]	1.00 (3.23) [190]	1.08 (3.40) [193]
Week 26 [*n*]	1.11 (3.47) [167]	0.94 (3.36) [168]
LS mean change (SE) from baseline [*n*]	0.11 (0.264) [190]	−0.10 (0.264) [193]
LS mean difference (SE) [95% CI] vs. Ly-Lis	0.21 (0.374) [−0.525 to 0.945]	
Mean 24-h plasma glucose from SMPG, mmol/L, mean (SD)		
Baseline [*n*]	10.07 (2.11) [201]	9.81 (2.05) [210]
Week 26 [*n*]	9.01 (2.17) [180]	9.00 (1.75) [189]
LS mean change (SE) from baseline [*n*]	−1.00 (0.137) [201]	0.91 (0.133) [210]
LS mean difference (SE) [95% CI] vs. Ly-Lis	−0.09 (0.191) [−0.464 to 0.287]	

LS means from MMRM with treatment group (SAR-Lis, Ly-Lis), randomization strata of screening HbA_1c_ (<8.0, ≥8.0%) and prior use of insulin lispro (Yes, No), visit (week 12, week 26), and treatment-by-visit interaction as fixed categorical effects, and baseline value and baseline value-by-visit interaction as continuous fixed covariates.

FPG, fasting plasma glucose; ITT, intent-to-treat; LS, least square; MMRM, mixed-effect model for repeated measures; SE, standard error; SMPG, self-monitored plasma glucose.

For the primary endpoint, the mean HbA_1c_ decreased similarly in both treatment groups from baseline to week 26, with the mean decrease in HbA_1c_ from baseline occurring mostly during the first 12 weeks of treatment (Fig. 2B). The LS mean (standard error [SE]) change in HbA_1c_ from baseline to week 26 was similar in the SAR-Lis (−0.92 [0.051]%) and Ly-Lis (−0.85 [0.051]%) groups (Table 2). The LS mean difference (SE; 95% CI) between the SAR-Lis group and the Ly-Lis group was −0.07% (0.072; −0.215 to 0.067). Noninferiority of SAR-Lis versus Ly-Lis was demonstrated, as the upper bound of the two-sided 95% CI of the difference between SAR-Lis and Ly-Lis was below the prespecified noninferiority margin of 0.3%. The inverse noninferiority of Ly-Lis versus SAR-Lis was also demonstrated. At week 26, similar percentages of randomized patients reached HbA_1c_ target <7% (SAR-Lis: 42.3%; Ly-Lis: 40.5%) and HbA_1c_ target ≤6.5% (SAR-Lis: 27.3%; Ly-Lis: 24.2%).

Mean FPG also decreased similarly in both groups (Fig. 2C). The LS mean change (SE) from baseline in FPG to week 26 was similar in the SAR-Lis group (−0.62 [0.176] mmol/L) and the Ly-Lis group (−0.67 [0.176] mmol/L). The LS mean (SE; 95% CI) difference between SAR-Lis and Ly-Lis was 0.06 (0.249; −0.430 to 0.547) mmol/L (Table 2). The mean seven-point SMPG profiles in both treatment groups improved at all time points at week 26 compared with baseline (Fig. 2D). The LS mean difference (SE; 95% CI) for SAR-Lis versus Ly-Lis for postprandial glucose excursions at breakfast, lunch, and dinner were −0.48 (0.328; −1.127 to 0.164), −0.05 (0.357; −0.749 to 0.655), and 0.21 (0.374; −0.525 to 0.945) mmol/L, respectively (Table 2). Mean 24-h PG values at baseline and week 26 were also similar between the two groups, with an LS mean (SE; 95% CI) difference of −0.09 (0.191; −0.464 to 0.287) mmol/L (Table 2).

In the SORELLA 2 study, the subgroup analyses based on baseline data in obese patients with BMI ≥30 versus <30 kg/m^2^, by diabetes duration (≥10 years vs. <10 years), in the elderly (<65 years vs. ≥65 to <75 years), and by ethnicity were consistent with the total patient population.

### Safety

The percentage of patients with at least one hypoglycemia event (regardless of the category) reported at any time of the day was similar in the SAR-Lis (68.4%) and Ly-Lis (74.6%) groups, and similar percentages of patients reported nocturnal hypoglycemia (00:00–05:59 h) in the SAR-Lis group and the Ly-Lis group (regardless the category) (Table 3). The occurrence of severe hypoglycemia, although imbalanced, was very low (<0.1 per patient-year) in both treatment groups. Severe hypoglycemia was reported in six patients (2.4%) in the SAR-Lis group and four patients (1.6%) in the Ly-Lis group, and nocturnal severe hypoglycemia in two patients (0.8%) and none, respectively. In all other predefined categories of hypoglycemia, the percentages of patients were similar with SAR-Lis and Ly-Lis.

**Table 3.** Summary of Hypoglycemia Events—Safety Population

	*All hypoglycemia*		*Nocturnal hypoglycemia (00:00–05:59)*	
*Type of hypoglycemia*	*SAR-Lis (*N* = 253)*	*Ly-Lis (*N* = 252)*	*SAR-Lis (*N* = 253)*	*Ly-Lis (*N* = 252)*
Total patient years	118.69	121.23	118.69	121.23
Any hypoglycemia				
Patients, *n* (%)	173 (68.4)	188 (74.6)	74 (29.2)	85 (33.7)
No. of events (rate/patient-year)	1992 (16.78)	2254 (18.59)	264 (2.22)	304 (2.51)
Severe hypoglycemia				
Patients, *n* (%)	6 (2.4)	4 (1.6)	2 (0.8)	0
No. of events (rate/patient-year)	9 (0.08)	4 (0.03)	2 (0.02)	0
Documented symptomatic hypoglycemia ≤3.9 mmol/L (70 mg/dL)				
Patients, *n* (%)	152 (60.1)	167 (66.3)	64 (25.3)	69 (27.4)
No. of events (rate/patient-year)	1345 (11.33)	1478 (12.19)	204 (1.72)	216 (1.78)
Documented symptomatic hypoglycemia <3.0 mmol/L (54 mg/dL)				
Patients, *n* (%)	73 (28.9)	69 (27.4)	21 (8.3)	20 (7.9)
No. of events (rate/patient-year)	193 (1.63)	196 (1.62)	33 (0.28)	33 (0.27)
Asymptomatic hypoglycemia ≤3.9 mmol/L (70 mg/dL)				
Patients, *n* (%)	89 (35.2)	94 (37.3)	20 (7.9)	24 (9.5)
No. of events (rate/patient-year)	409 (3.45)	598 (4.93)	26 (0.22)	49 (0.40)
Asymptomatic hypoglycemia <3.0 mmol/L (54 mg/dL)				
Patients, *n* (%)	26 (10.3)	32 (12.7)	3 (1.2)	3 (1.2)
No. of events (rate/patient-year)	47 (0.40)	66 (0.54)	4 (0.03)	3 (0.02)
Severe and/or confirmed^a^ hypoglycemia ≤3.9 mmol/L (70 mg/dL)				
Patients, *n* (%)	169 (66.8)	183 (72.6)	73 (28.9)	79 (31.3)
No. of events (rate/patient-year)	1907 (16.07)	2154 (17.77)	248 (2.09)	278 (2.29)
Severe and/or confirmed^a^ hypoglycemia <3.0 mmol/L (54 mg/dL)				
Patients, *n* (%)	89 (35.2)	84 (33.3)	26 (10.3)	22 (8.7)
No. of events (rate/patient-year)	271 (2.28)	277 (2.28)	40 (0.34)	39 (0.32)

*n* (%), number and percentage of patients with at least one treatment-emergent hypoglycemia event.

^a^ Severe and/or confirmed hypoglycemia = severe and/or confirmed by plasma glucose ≤3.9 mmol/L (70 mg/dL) or <3.0 mmol/L (54 mg/dL).

Most hypoglycemia was observed between 7 a.m. and midnight with small peaks around each meal (Supplementary Fig. S2). There were no relevant differences in event rates between the two groups. The event rate of any hypoglycemia was similar in both treatment groups with 16.78 events per patient-year of exposure in the SAR-Lis group and 18.59 events per patient-year of exposure in the Ly-Lis group (Table 3). The nocturnal hypoglycemia event rate was low and also similar in the SAR-Lis and Ly-Lis groups, being 2.22 and 2.51 events per patient-year of exposure, respectively. The only category of hypoglycemia where a difference between the two groups was observed was severe hypoglycemia where the annualized event rate was 0.08, with nine events reported in six patients in the SAR-Lis group, and 0.03, with four events reported in four patients in the Ly-Lis group. The higher rate in the SAR-Lis group was due to one patient who reported four events of severe hypoglycemia. No factors were identified that may have contributed to this patient's frequent low blood glucose. Most patients with severe hypoglycemia had prompt recovery further to corrective treatment. Serious TEAEs involving hypoglycemia were reported in two patients (0.8%) in each group.

A similar percentage of patients in the SAR-Lis and Ly-Lis group reported a TEAE (SAR-Lis, 46.6%; Ly-Lis, 42.9%; Table 4), the most common of which was nasopharyngitis (SAR-Lis, 4.0%; Ly-Lis, 2.0%). Serious TEAEs were reported in a lower percentage of patients in the SAR-Lis group (5.5% [14 patients]) than in the Ly-Lis group (10.7% [27 patients]). Events were distributed over a variety of system organ classes (SOCs) with an incidence no higher than 1.6% (four patients) in any SOC in any treatment group, except for cardiac disorders, which was reported in 1.2% (three patients) in the SAR-Lis group and 4.4% (11 patients) in the Ly-Lis group. Seven patients (2.8%) in the SAR-Lis group and six patients (2.4%) in the Ly-Lis group reported TEAEs leading to permanent discontinuation of the investigational drug. A total of three deaths occurred during the study period: one death (0.4%) in the SAR-Lis group due to a cardio-respiratory arrest and two deaths (0.8%) in the Ly-Lis group due to cardiopulmonary failure and bladder cancer with metastasis. One additional patient in the Ly-Lis group died after the end of the study of unknown causes. The deaths were considered not related to IMP.

**Table 4.** Summary of Injection Site and Hypersensitivity Reactions and Adverse Events (Safety Population), and Anti-Insulin Antibodies (AIA Population)

*Safety population*	*SAR-Lis (*N* = 253)*	*Ly-Lis (*N* = 252)*
Any injection site reaction	1 (0.4)	4 (1.6)
Any hypersensitivity reactions	10 (4.0)	9 (3.6)
Patients with any TEAE	118 (46.6)	108 (42.9)
Patients with any treatment-emergent SAE	14 (5.5)	27 (10.7)
Patients with any TEAE leading to death	1 (0.4)	2 (0.8)
Patients with any TEAE leading to permanent IMP discontinuation	7 (2.8)	6 (2.4)

*AIA population*	*SAR-Lis (*N* = 245)*	*Ly-Lis (*N* = 248)*
Patients with AIA positive at baseline, *n* (%)	60/245 (24.5)	63/248 (25.4)
Patients with ≥4-fold increase in titer (treatment boosted), *n* (%)	12/60 (20.0)	8/63 (12.7)
Patients with AIA negative or missing at baseline, *n* (%)	185/245 (75.5)	185/248 (74.6)
Patients newly positive postbaseline (treatment induced), *n* (%)	34/185 (18.4)	28/185 (15.1)
Patients with at least one positive AIA sample (prevalence),^a^*n* (%)	94/245 (38.4)	91/248 (36.7)
Patients with treatment-emergent AIA (incidence),^b^*n* (%)	46/245 (18.8)	36/248 (14.5)
No. (%) of patients AIA positive at week 26	68/221 (30.8%)	66/226 (29.2%)

Data are *n* (%) = number and percentage of patients with at least one TEAE.

^a^ Prevalence: patients AIA positive at baseline plus those with treatment-induced AIAs.

^b^ Incidence: patients with treatment-boosted or treatment-induced AIAs (i.e., patients with treatment-emergent AIAs).

AIA, anti-insulin antibodies; IMP, investigational medicinal product; SAE, serious adverse event; TEAE, treatment-emergent adverse event.

The mean increase in body weight from baseline to week 26 was similar in the SAR-Lis (+1.35 kg) and Ly-Lis (+1.32 kg) groups. No clinically meaningful changes from baseline were observed in clinical laboratory and hematology parameters, and no relevant differences between the two treatment groups occurred.

### Immunogenicity

Similar percentages of patients in both treatment groups were positive for AIA at baseline (SAR-Lis, 24.5%, and Ly-Lis, 25.4%) (Table 4). The percentage of patients with a treatment-emergent AIA response (i.e., treatment-boosted or treatment-induced AIAs; incidence) was 18.8% (46/245) in the SAR-Lis group and 14.5% (36/248) in the Ly-Lis group. Over the 6-month period, percentages of patients positive for AIA slightly increased in both treatment groups: 30.8% of SAR-Lis patients and 29.2% of Ly-Lis patients at week 26. Similar percentages of patients in the SAR-Lis group (38.4%) and Ly-Lis group (36.7%) were positive for AIAs at least at one time point between baseline and month 6 (prevalence). Cross-reactivity with human insulin, insulin glargine, and insulin glargine M1 metabolite was high (80%–90%) and consistent between treatment groups. No relationship was observed between the individual maximal AIA titers and the change in total insulin dose, HbA_1c_, hypoglycemia, injection site, and hypersensitivity reactions.

A low number of patients reported hypersensitivity reactions (SAR-Lis, 10 [4.0%] and Ly-Lis, 9 [3.6%]) and very few patients reported injection site reactions (SAR-Lis, 1 [0.4%] and Ly-Lis, 4 [1.6%]) (Table 4). Most events were mild or moderate in intensity. All resolved while treatment was ongoing, with the exception of one event in the SAR-Lis group (dermatitis contact) and three events in the Ly-Lis group (one event each of dermatitis, rash, and facial edema). Out of the 24 potential hypersensitivity reactions reported in either treatment group, only four events (seasonal allergy, contact dermatitis, allergy to arthropod bite, and allergic rhinitis) in the SAR-Lis group and three events (pruritus [two] and mouth swelling) in the Ly-Lis group were adjudicated as allergic reaction by the ARAC; the two events of pruritus in the Ly-Lis group were considered related to IMP.

## Discussion

Insulins approved as biosimilars or follow-on biologics expand the number of insulin brands available for those with diabetes and may have the potential to reduce diabetes treatment cost. Indeed, these products are usually marketed at a lower price than the originator product. This was first observed after the market launch of erythropoietin biosimilars.

In this study, we report on the pharmacological characteristics of SAR-Lis insulin, a biosimilar of Ly-Lis insulin with an identical amino acid sequence.^12^ Similar efficacy in terms of changes in HbA_1c_ levels was noted between SAR-Lis and Ly-Lis at the primary endpoint at week 26, and noninferiority of SAR-Lis to Ly-Lis and of Ly-Lis to SAR-Lis was demonstrated in accordance with the guidance from the US Food and Drug Administration (FDA) and European Medicines Agency (EMA).^4–8^ The FPG and seven-point SMPG profiles were similar between treatment groups except for some small differences at certain time points, which were not considered clinically relevant. All hypoglycemic events and event rates were similar in both treatment groups, across all ADA categories, except for a higher rate of severe hypoglycemia with SAR-Lis than Ly-Lis due to one patient who reported four events. The general safety profile (percentages of patients with any TEAE, serious TEAEs, or TEAEs leading to study medication discontinuation, as well as type of TEAEs) was also similar between treatment groups. Although a slightly higher percentage of patients with treatment-emergent AIA was observed in the SAR-Lis group, no impact on efficacy and safety was observed in either group.

The original registration studies with Ly-Lis showed altered efficacy of Ly-Lis in obese patients; thus the FDA mandated postmarketing studies in obese patients after the product was approved in 1996.^13^ In the SORELLA 2 study, the subgroup analyses based on baseline data in obese patients with BMI ≥30 kg/m^2^ versus <30 kg/m^2^, by diabetes duration (≥10 years vs. <10 years), in the elderly (<65 years vs. ≥65 to <75 years), and by ethnicity were consistent with the total patient population. In particular, the incidence of hypoglycemia was comparable between treatments for the subgroups, including incidence of severe hypoglycemia.

The study included a study population that is largely adult white Caucasian with small numbers of blacks and Asians. Caution should be taken when extending the results to other ethnic populations or subgroups, as the study was not powered for them. The open-label study design was chosen as the prefilled, disposable pen injection devices for SAR-Lis and Ly-Lis could not be made indistinguishable. However, outcome assessments were determined based on objectively collected data determined by central laboratories blinded to the study treatment.

We conclude that SAR-Lis and Ly-Lis when used for 6 months in combination with GLA-100 provided effective and similar glucose control in patients with T2DM. SAR-Lis and Ly-Lis had similar safety and immunogenicity profiles and no specific safety concerns were observed.

## Supplementary Material

Supplemental data

Supplemental data
