# Supplementary figures and images for: Efficacy and Safety of Biosimilar SAR342434 Insulin Lispro in Adults with Type 2 Diabetes, Also Using Insulin Glargine: SORELLA 2 Study
Source: Diabetes Technol Ther. 2018 Jan 1;20(1):49–58. doi: 10.1089/dia.2017.0281 (PMC5770084; doi:10.1089/dia.2017.0281)

Supplementary Data

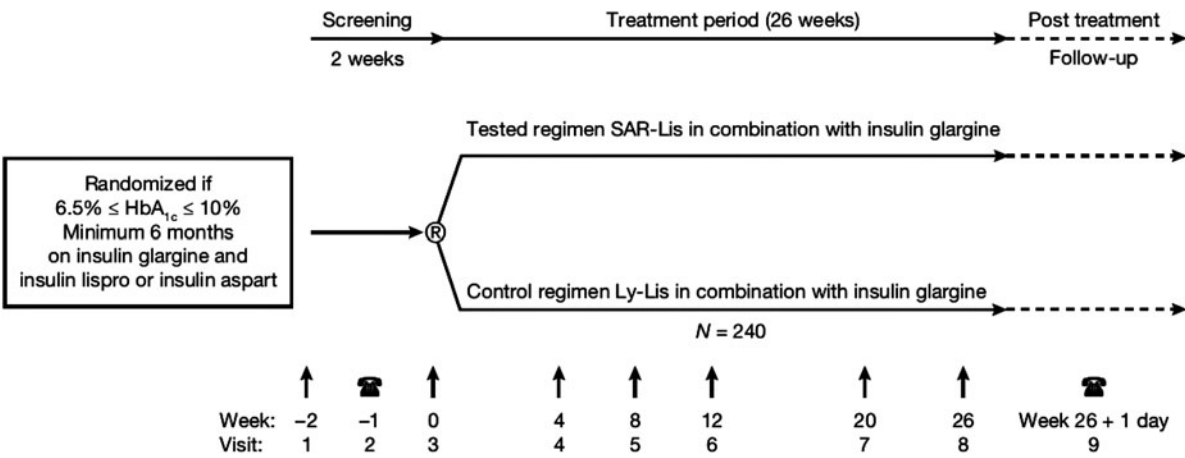

SUPPLEMENTARY FIG. S1. Study design.

Supplement: Supplemental data [file Supp_Figure1.pdf]
