# Supplementary material for: Efficacy and Safety of Biosimilar SAR342434 Insulin Lispro in Adults with Type 2 Diabetes, Also Using Insulin Glargine: SORELLA 2 Study
Source: Diabetes Technol Ther. 2018 Jan 1;20(1):49–58. doi: 10.1089/dia.2017.0281 (PMC5770084; doi:10.1089/dia.2017.0281)
Supplement: Supplemental data [file Supp_Figure2.pdf]

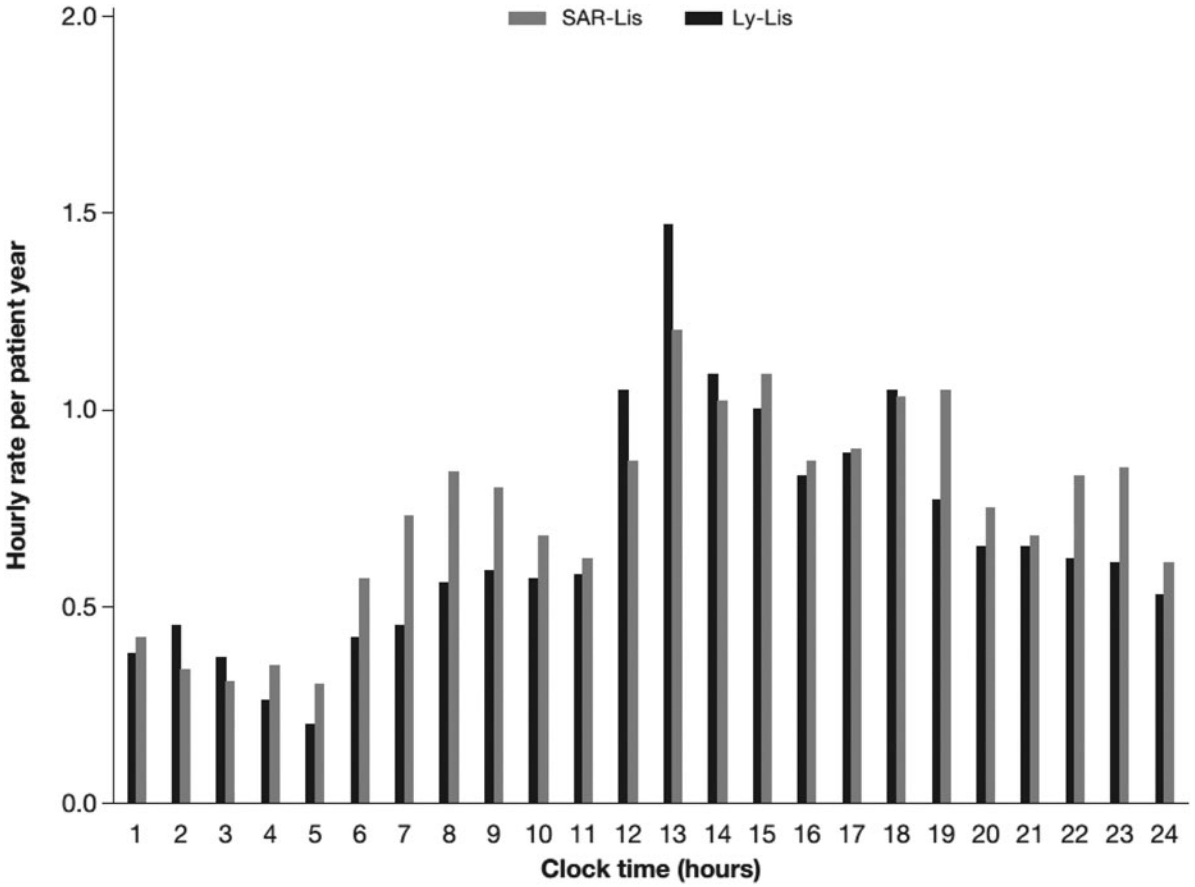

SUPPLEMENTARY FIG. S2. Hourly rate of severe and/or confirmed hypoglycemia ≤3.9 mmol/L (70 mg/dL) per patient-year during the 6-month on-treatment period—safety population.
